# Enhanced Bladder Regeneration with Adipose-Derived Stem Cell-Seeded Silk Fibroin Scaffolds: A Comparative Analysis

**DOI:** 10.3390/biomimetics10020093

**Published:** 2025-02-07

**Authors:** Hanan Hendawy, Ahmed Farag, Asmaa Elhaieg, Elsayed Metwllay, Kazumi Shimada, Ahmed Elfadadny, Ryou Tanaka

**Affiliations:** 1 Department of Veterinary Surgery, Faculty of Veterinary Medicine, Tokyo University of Agriculture and Technology, Fuchu 183-8509, Japan; hanan_attia@vet.suez.edu.eg (H.H.); ahmedfarag9331@gmail.com (A.F.);; 2 Department of Veterinary Surgery, Faculty of Veterinary Medicine, Suez Canal University, Ismailia 41522, Egypt; 3 Department of Surgery, Anesthesiology, and Radiology, Faculty of Veterinary Medicine, Zagazig University, Zagazig 44519, Egypt; 4 Department of Cytology and Histology, Faculty of Veterinary Medicine, Suez Canal University, Ismailia 41522, Egypt; elsayedalalfey@vet.suez.edu.eg; 5 Department of Animal Internal Medicine, Faculty of Veterinary Medicine, Damanhur University, Damanhour 22511, Egypt; ahmed.elfdadny@vetmed.dmu.edu.eg

**Keywords:** silk fibroin, adipose-derived stem cells, urothelium, muscle, bladder regeneration

## Abstract

Effective bladder reconstruction remains a significant challenge in urology, particularly for conditions requiring partial or complete bladder replacement. In this study, the efficacy is evaluated of two types of scaffolds, silk fibroin (SF) and adipose-derived stem cells (ADSCs-SF), in promoting bladder regeneration and their associated outcomes. A rat model was used to compare the surgical outcomes and morphological recovery of bladder tissues implanted with SF and ADSCs-SF scaffolds. Post-operative recovery, including voiding ability and complication rates, was assessed. The morphological and histological changes of the regenerated bladder tissue were evaluated at multiple time points (2, 4, 8, and 12 weeks) using gross tissue analysis, histometric assessments, and immunohistochemical staining. Both scaffold types demonstrated successful integration into the bladder wall with no significant differences in body weight or voiding issues. The SF scaffold group exhibited graft shrinkage and a 41.6% incidence of bladder calculus formation. In contrast, the ADSCs-SF scaffold facilitated superior morphological restoration, with bladder tissue progressively adopting a more normal shape and no incidence of bladder calculus. Histological analysis revealed that the ADSCs-SF scaffold significantly promoted the regeneration of a more organized urothelium layer and smooth muscle tissue. It also resulted in higher vessel density and reduced infiltration of inflammatory cells when compared to the SF scaffold alone. Additionally, the ADSCs-SF group exhibited enhanced expression of key markers, including uroplakin III, a urothelial marker, and α-SMA, a smooth muscle cell marker. These findings suggest that the ADSCs-SF scaffold not only supports the structural integrity of the bladder but also improves tissue regeneration and reduces adverse inflammatory responses, offering a promising approach for bladder repair and reconstruction.

## 1. Introduction

Bladder reconstruction is a crucial procedure used to treat a variety of conditions affecting the urinary tract, including congenital abnormalities like bladder exstrophy, acquired issues such as bladder cancer, and functional disorders like neurogenic bladder dysfunction [1]. At present, this procedure is considered one of the most complex and demanding surgical interventions in the field of urology. However, advances in tissue engineering techniques show great promise for bladder repair and reconstruction. These innovative approaches offer a potential solution for addressing a wide range of complications and side effects often associated with traditional surgical methods, potentially improving patient outcomes and reducing recovery times. With ongoing research and development, tissue engineering could revolutionize the treatment of bladder-related conditions, providing a more effective and less invasive alternative to conventional procedures [2,3].

Recent studies have highlighted the growing use of various scaffolds, primarily derived from naturally occurring materials or synthetic polymers in addition to hybrid materials that have been used in bladder repair [4,5]. By incorporating advanced materials and innovative design features, these scaffolds not only promote better integration with the surrounding tissues but also contribute to reduced complications, faster recovery, and improved long-term functionality of the bladder. As the research continues, these scaffold-based techniques are expected to revolutionize the field of bladder repair and reconstruction [6,7].

Silk fibroin (SF) is a distinctive natural fibrous protein recognized for its outstanding properties, making it highly valuable in various biomedical applications. It boasts remarkable structural strength and elasticity, which provide durability and flexibility in tissue engineering [8,9]. Additionally, SF offers versatile processing capabilities, allowing it to be fabricated into various forms such as films, scaffolds, and hydrogels [10]. Unfortunately, despite its many advantages, the simple and compact structure of SF poses a challenge in terms of its degradability. The material’s slow degradation rate can result in its persistence within the body, which may increase the risk of complications, such as the formation of urinary stones. Therefore, careful consideration and modification of its degradation properties are necessary to mitigate such risks and ensure its safe and effective use in bladder repair and reconstruction [11,12].

For optimal urinary bladder regeneration, an ideal scaffold must fulfill several essential criteria. It should provide a suitable three-dimensional (3-D) structure that mimics the natural architecture of the bladder, enabling proper cellular organization and tissue formation. The scaffold must also support the specific microenvironmental needs of the various cell types in the bladder wall, including urothelial cells, smooth muscle cells, and endothelial cells [9,13]. Additionally, it must ensure the maintenance of the bladder’s barrier function, protecting against harmful substances and preventing infections. Finally, the scaffold should preserve the structural integrity of the bladder, ensuring it can withstand the mechanical stresses associated with normal bladder function. Meeting these requirements is crucial for promoting effective bladder regeneration and long-term functionality [14,15].

The seeded scaffold strategy has several drawbacks, including a long preparation time, higher costs, and the risk of contamination, all of which limit its clinical use [16,17]. In contrast, the cell-free approach is more suited for large-scale clinical applications. Off-the-shelf approved patch products can be used, and the native cells recruited by the patch can help regenerate tissue in vivo. Stem cell therapy offers a promising solution for improving the regeneration of tissue-engineered bladders. Various cell types have been suggested, such as pluripotent stem cells, human umbilical mesenchymal stem cells, urothelial, and smooth muscle cells [18]. In this model, ADSCs were chosen because they can be obtained in large quantities with minimal risk to the patient. Furthermore, ADSCs have the ability to differentiate into various lineages, including adipogenic, osteogenic, and chondrogenic cells [19,20]. By capitalizing on the advantages of ADSCs—such as easy availability, low extraction-related morbidity, and long-term survival in the urinary tract [18]—and leveraging the unique properties of ADSCs, we seek to develop more effective and sustainable solutions for bladder regeneration, improving the clinical outcomes and minimizing the complications associated with traditional methods.

## 2. Materials and Methods

All animal experiments were carried out in strict accordance with the institutional guidelines, ensuring ethical treatment and proper care throughout this study. The procedures followed were approved by the Animal Care and Use Committee of Tokyo University of Agriculture and Technology (approval no. R04-156), which is responsible for overseeing the welfare of animals used in research.

### 2.1. Animals

The effectiveness of SF and ADSCs-SF scaffolds was evaluated in a bladder augmentation model using adult female Sprague Dawley rats (8 weeks old), following previously established protocols [21]. The rats were separated into the following three groups: the sham group (*n* = 3), the SF group (*n* = 12), and the ADSCs-SF group (*n* = 12). To assess the long-term impact of the scaffolds, rats in the SF and ADSCs-SF groups were euthanized at 2, 4, 8, and 12 weeks after implantation. In contrast, the sham group, which did not receive scaffold implants, was sacrificed at the 12-week time point. This design allowed for a thorough evaluation of tissue response, scaffold integration, and overall efficacy at various stages post-implantation.

### 2.2. Fabrication of SF Scaffold

Silk fiber was obtained from silk threads by reeling and degumming the cocoons of Bombyx mori [22]. The natural cocoons were subjected to two rounds of degumming using a 0.5 wt.% sodium carbonate (Na_2_CO_3_) solution at 100 °C for 30 min, followed by rinsing with deionized water to remove the sericin, as described in previous research [23]. The degummed silk fibers were then dissolved in a 9.0 M lithium bromide (LiBr) solution at 40 °C for 2 h. The regenerated SF solution was diluted and dialyzed against deionized water at 10 °C for 3 days using a cellulose semi-permeable membrane with a molecular weight cutoff of 14,000. After dialysis, the SF solution was concentrated to 33 wt.% by forced airflow at 10 °C. The solution concentration was determined by weight measurement [24]. Electrospinning was carried out as described in prior studies [25]. Briefly, the SF solution was loaded into a 2.5 mL syringe with a 6 G needle (0.6 mm inner diameter) as the spinneret. Electrospinning occurred at a voltage of 19 kV and a flow rate of 19 μL/min for 106 min. An aluminum foil, grounded and positioned 16 cm from the needle, was used to collect the randomly deposited fibers.

For 20 min at room temperature following spinning, SF electrospun nanofibers were immersed in 80% *v*/*v* ethanol to cause a conformational transition. The remaining ethanol was then removed by washing them in distilled water at 37 °C for an entire night with gentle stirring. They were then dried at room temperature for twenty-four hours at 100% humidity. Finally, ethylene oxide gas was used to sterilize the scaffolds. They had the following dimensions for scaffold implantation: width 10 mm, length 10 mm, and thickness 100 mm.

### 2.3. ADSCs Culture and Seeding

Renal ADSCs were isolated and characterized following our previously established protocols [19,20]. The ADSCs were cultured in Dulbecco’s Modified Eagle Medium (DMEM) supplemented with 10% fetal bovine serum (FBS). The cells were incubated in a humidified atmosphere chamber maintained at 37 °C with 5% carbon dioxide. The ADSCs were allowed to expand until the required cell numbers were achieved. After expansion, the cells were trypsinized, washed, and collected as a pellet. To ensure the preservation of their multipotency, the ADSCs were expanded for a total of three passages. The prepared SF scaffolds were set up on scaffold holders and then placed in the 24-well plate following an hour-long UV disinfection. Subsequently, the ADSCs were seeded onto the SF scaffolds by placing 50 μL of a cell suspension (10 × 10^7^ cells/mL) onto the SF layer, and cells were cultured for 4 h at 37 °C to allow cell attachment to the scaffolds. During this incubation step, 3 mL of DMEM containing 10% FBS was gradually added every 15 min to keep them wet. After that, 20 mL of the culture medium was added and SF scaffolds seeded with the ADSCs were cultured at 37 °C in 5% CO_2_ and 95% humidity for 3 days before implantation. The culture medium was replaced daily for the next 3 days following a previous protocol [26].

### 2.4. Surgical Technique for Rat Bladder Augmentation

The rats were anesthetized using isoflurane inhalation anesthesia. Initially, they were placed in an induction chamber with 5% isoflurane and room air until they became laterally recumbent. Once anesthetized, they were transferred to a circulating warm-water blanket to maintain body temperature. Anesthesia was maintained with 2.0% to 2.5% isoflurane and oxygen through a semi-closed non-rebreathing system. Anesthetic depth was monitored by assessing the toe pinch reflex and respiratory rate. The ventral abdomen and vulva areas were shaved and sterilized with 70% ethanol to prepare for the procedure. The rat bladder augmentation model was conducted following a previously described protocol with minor modifications [27].

A sterile ventral midline laparotomy incision was performed, and the peritoneum was carefully dissected to reveal the bladder. The bladder’s ventral ligament was then cut, and the apex of the bladder was held at the cranial part of the ventral ligament to ensure proper stabilization. A 1.5 cm longitudinal cystotomy incision was made on the bladder wall using fine scissors, creating a defect in the bladder.

**Figure 1:**
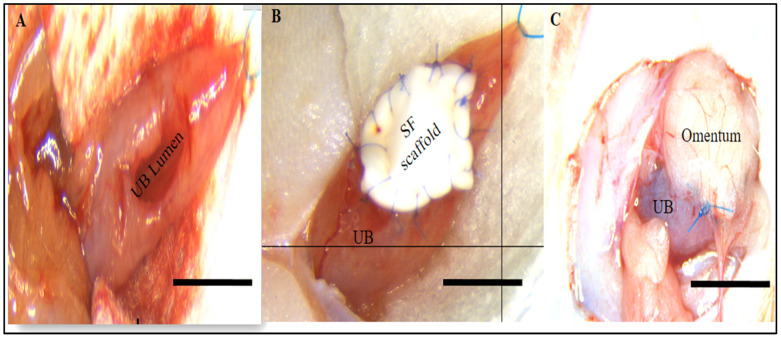
Representative images illustrating the surgical procedure for bladder augmentation using silk fibroin (SF) scaffolds. (**A**) The bladder lumen is fully exposed. (**B**) The SF scaffold is integrated into the defect on the bladder wall. (**C**) The surgical site is covered with the omentum. UB (urinary bladder); SF (silk fibroin). Scale bar: 1 cm.

A square piece of either the SF or ADSCs-SF scaffold (10 mm × 10 mm) was carefully positioned into the bladder defect site. The scaffold was fixed in place using 8-0 polyglactin absorbable sutures (Ethicon; Johnson & Johnson Services, Inc., New Brunswick, NJ, USA), ensuring accurate attachment and alignment. To verify the integrity of the procedure, a watertight seal was formed by filling the bladder with sterile saline through a 30-gauge hypodermic needle. The scaffold was then covered with omentum for added protection and support, aiding in the healing process (Figure 1). This approach ensured the scaffold remained securely in place, promoting effective bladder augmentation.

Lastly, the linea alba was closed with 5-0 polydioxanone sutures, and swaged onto an RB-1 taper needle, in a simple continuous pattern. The skin edges were approximated using both subcuticular and subcutaneous sutures to ensure proper closure and minimize scarring. Following the surgery, the rats were allowed to recover from anesthesia by discontinuing isoflurane and breathing room air.

Post-operative care was provided to support the animals’ recovery. This included the administration of food and water, as well as enrofloxacin (10 mg/kg, intramuscularly, once daily for 5 days; Bayer, Berlin, Germany) to prevent infection. Additionally, carprofen (0.1 mg/kg, intramuscularly, once daily for 3 days; Pfizer, New York, NY, USA) was given to manage pain and inflammation.

The rats were then monitored and assessed independently at various time points, as follows: 2, 4, 8, and 12 weeks following the implantation procedure. These assessments were designed to evaluate the healing progress, any potential complications, and the long-term outcomes of the bladder augmentation surgery.

### 2.5. Histological and Immunohistochemical Evaluation of Regenerative Processes in the Reconstructed Bladder Wall

Hematoxylin and eosin (H&E) staining was used to assess the presence and organization of key tissue components, such as urothelial layers, muscle bundles, and blood vessels, in the newly constructed bladder. The paraffin-embedded sections were first deparaffinized in xylene and then rehydrated through a graded series of ethanol solutions. The sections were then stained with hematoxylin for 5 min, and counterstained with eosin for 2 min. Following staining, the slides were dehydrated through a series of ethanol solutions, cleared in xylene, and mounted with a coverslip for microscopic examination.

For immunohistochemistry, tissue sections were dewaxed in xylene and subjected to heat-induced epitope retrieval using citrate buffer solution (NB900-62075; Novus Biologicals, Centennial, CO, USA) for 20 min. Endogenous peroxidase activity was inhibited by incubating the sections with 0.3% hydrogen peroxide for 10 min at room temperature. To prevent non-specific binding, the sections were blocked with 5% bovine serum albumin in PBS for 20 min before applying the primary antibodies. Urothelial cells in the reconstructed tissue were identified using an anti-uroplakin III antibody (ab78196, 1:200; Abcam, Cambridge, UK), while smooth muscle cells (SMCs) were detected with an anti-alpha smooth muscle actin antibody (α-SMA, ab7817, 1:200; Abcam). Horseradish peroxidase-conjugated secondary antibodies were then used. A freshly prepared 3,3′-diaminobenzidine solution served as the chromogen for colorimetric detection. Immunostaining densities were quantified using Fiji software in ImageJ (1.8.0-345), with nuclear staining employed as an internal control. The average intensity of the protein signal within a 500 µm^2^ region of interest was normalized to the average intensity of the control.

### 2.6. Statistical Analysis

All data are expressed as the mean ± standard deviation (S.D.). Statistical analyses were conducted using GraphPad Prism software version 6 (GraphPad Software, Inc., La Jolla, CA, USA). The data were analyzed using one-way analysis of variance (ANOVA), followed by Tukey’s post hoc test to assess the differences between groups. Additionally, the Student’s *t*-test was employed to further evaluate statistical significance between specific groups. A *p*-value of less than 0.05 was considered statistically significant. The level of significance was indicated as * *p* < 0.05, ** *p* < 0.01, and *** *p* < 0.001, with the asterisks representing varying degrees of significance.

## 3. Results

### 3.1. Surgical Outcomes and Post-Operative Recovery

All animals successfully underwent the surgical procedures and were able to void urine spontaneously following the operation. No instances of urine leakage were observed in any of the groups. Throughout this study, there were no significant differences in body weight, the occurrence of incontinence, urinary tract infections, the formation of diverticula, or any issues related to leakage or voiding abnormalities. All animals maintained normal urinary function post-surgery, with no evidence of complications or adverse effects. The recovery process was smooth and consistent across all groups, indicating the success and safety of the surgical intervention.

### 3.2. Assessment of Adhesions, Graft Shrinkage, and Morphological Changes in Bladder Regeneration Using SF and ADSCs-SF Scaffolds

**Figure 2:**
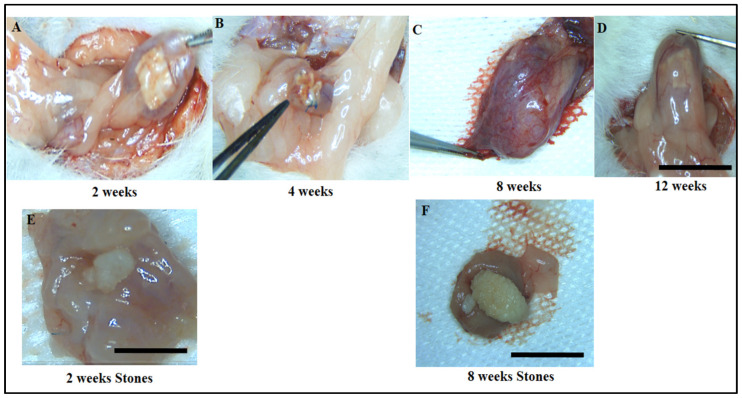
Macroscopic images showing the time-dependent progression of the SF scaffold implanted in the rat bladder at the following different time intervals: 2, 4, 8, and 12 weeks post-implantation. At 2 weeks, the SF scaffold was found to be well-integrated into the bladder wall (**A**). By 4 weeks post-implantation, noticeable changes in the scaffold’s structure were observed, including significant shrinkage (**B**). Simultaneously, the regenerated bladder tissue began to take shape, forming a pouch-like, irregular structure (**C**). At 8 and 12 weeks, this pouch-like structure became more pronounced, with the regenerated bladder tissue showing further maturation and differentiation (**D**). Gross morphological examination revealed the formation of bladder calculi inside the bladder lumen at both 2 weeks (**E**) and 8 weeks (**F**) in the SF group. Scale bar: 1 cm.

Gross tissue evaluations revealed minimal adhesions between the bladder and the adjacent omentum. After careful and precise dissection of these adhesions, only minor scar tissue formation was observed, suggesting minimal disruption to surrounding tissues. In the SF scaffold group, a noticeable degree of graft shrinkage was evident at 4 weeks post-implantation, particularly when compared to the earlier 2-week period (Figure 2A,B). This shrinkage was accompanied by a distinct change in the appearance of the regenerated bladder tissue, which developed a pouch-like, irregular shape between 8 and 12 weeks post-implantation (Figure 2C,D). Furthermore, an unexpected complication arose in the SF scaffold group, where bladder calculus formation was observed in 41.6% of the cases, with five out of twelve subjects developing bladder stones (Figure 2E,F). This suggests that while the SF scaffold may have potential for bladder regeneration, it could also predispose the tissue to complications such as the formation of calculi. The results clearly showed that the ADSCs-SF scaffold played a crucial role in progressively promoting the morphological restoration of the regenerated bladder.

**Figure 3:**
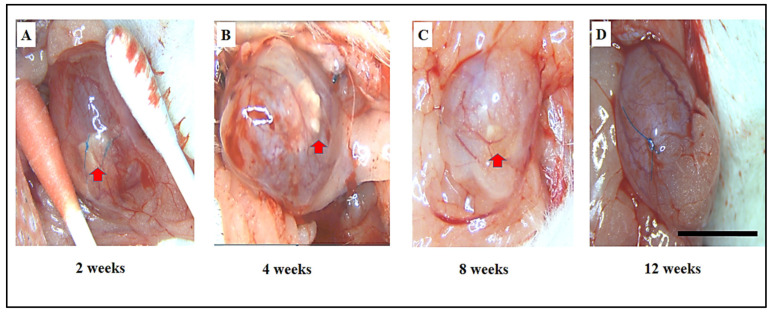
Macroscopic images showing the time-dependent progression of the ADSCs-SF scaffold implanted in the rat bladder at various time points, as follows: 2, 4, 8, and 12 weeks post-implantation. At 2 weeks post-implantation, the scaffold began to show initial signs of integration and tissue response, though the surface remained irregular (**A**). By 4 weeks, the surface of the regenerated bladder tissue started to smooth out, though it still appeared somewhat uneven and irregular (**B**). At 8 and 12 weeks, a notable improvement in the tissue’s shape was observed, with the bladder adopting a more oval, structurally coherent form, which closely resembled the normal bladder morphology (**C**,**D**). Red arrows refer to the SF scaffold within the bladder. Scale bar: 1 cm.

At the two-week mark following implantation (Figure 3A), the bladder displayed an irregular and uneven surface, indicative of early-stage healing. By four weeks post-implantation (Figure 3B), the bladder had started to adopt a more oval shape, signaling a positive trend toward recovery. By the 8- to 12-week period (Figure 3C,D), the bladder closely resembled the natural shape of the sham group, exhibiting significant restoration. Furthermore, no bladder calculus formation was observed in the ADSCs-SF group, highlighting the scaffold’s potential to prevent such complications. These findings collectively suggest that the ADSCs-SF scaffold is notably more effective in promoting bladder morphological restoration, while also minimizing the risk of complications such as bladder calculus, in comparison to the SF-only scaffold.

### 3.3. Comparative Histological Analysis of Urothelium, Muscle Regeneration, Vessel Density, and Inflammatory Response in SF and ADSCs-SF Scaffold Implants

**Figure 4:**
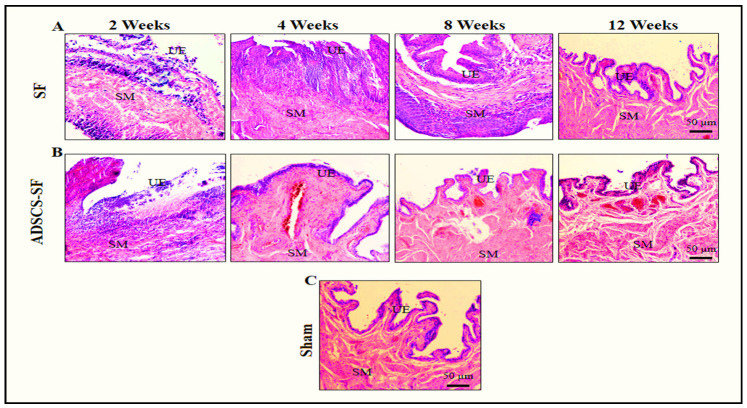
Histological examination of regenerated bladder tissue in the SF and ADSCs-SF groups using H&E staining. Panels (**A**,**B**) show the histological changes at different time points for both scaffold groups, compared to the histological analysis of the bladder tissue from the sham group (**C**). UE (urothelium); SM (smooth muscle). Scale bars = 50 µm.

**Figure 5:**
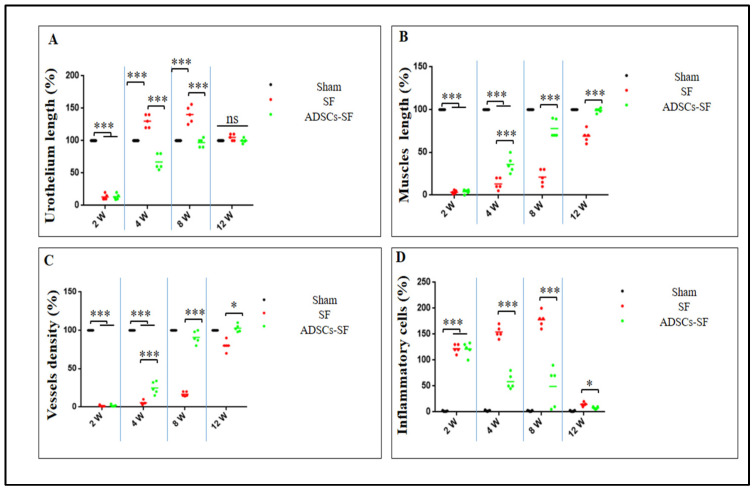
Quantitative assessment of the relative percentages of urothelium length (**A**), muscle length (**B**), vessel density (**C**), and inflammatory cell (**D**) presence in the regenerated bladder wall at 2, 4, 8, and 12 weeks after implantation of SF and ADSCs-SF scaffolds. Data are presented as mean ± standard deviation (SD). Statistical analysis was performed using one-way analysis of variance (ANOVA) with Tukey’s post hoc test, and pairwise comparisons were conducted using the Student’s *t*-test. Asterisks indicate statistically significant differences: * *p* < 0.05, *** *p* < 0.001.

To investigate the histological changes associated with the observed morphological developments, histometric analyses were conducted. These analyses highlighted the significant roles of the SF and ADSCs-SF scaffolds throughout various stages of bladder regeneration. Both scaffolds exhibited rapid biodegradation and successful incorporation into native tissue by 8 to 12 weeks in vivo (Figure 4 and Figure 5).

#### 3.3.1. Urothelium

At two weeks following implantation, both the SF and ADSCs-SF scaffolds showed the presence of a delicate, thin urothelial layer covering the luminal surface of the scaffolds (Figure 4A,B). This initial urothelial lining was relatively sparse but indicated early stages of tissue integration. Notably, a significant reduction in urothelium length was observed in both groups when compared to the sham group (Figure 5A), suggesting an impaired or delayed urothelial regeneration in the scaffold groups. This difference highlighted a potential issue with the initial cellular ingrowth or scaffold support at early time points.

Between weeks 4 and 8, the SF group demonstrated significant urothelial hyperplasia, characterized by a marked increase in urothelial cell proliferation. This condition led to a thicker, more expansive urothelial layer, but the tissue architecture remained somewhat disorganized and less uniform. In contrast, the ADSCs-SF group exhibited comparatively less hyperplasia during this time frame, with a slower but more controlled cellular response. Despite this, the ADSCs-SF group showed better tissue differentiation and organization within the urothelium, suggesting that the incorporation of ADSCs may have promoted a more orderly regeneration process.

By 12 weeks post-implantation, both scaffold types had developed a multilayered urothelium at the implantation sites, indicating more mature tissue formation. However, no significant differences in urothelium length were observed between the SF and ADSCs-SF groups at this stage, suggesting that both scaffolds ultimately supported similar levels of urothelial growth in terms of overall thickness. Despite this similarity, the ADSCs-SF group stood out for its more structured and organized multilayered urothelial architecture (Figure 4). The layered urothelium in the ADSCs-SF group appeared more cohesive, with well-differentiated cellular layers that suggested better functional and structural integration compared to the SF scaffold alone. This finding indicates that the inclusion of ADSCs within the scaffold may have a positive influence on the quality of tissue regeneration, leading to a more organized and potentially more resilient urothelial layer.

#### 3.3.2. Smooth Muscle Cells

At two weeks post-implantation, both scaffold groups showed a marked and highly significant reduction in muscle length compared to the control group (Figure 5B). This early finding indicated that muscle regeneration was delayed or hindered in both experimental groups during the initial stages following implantation. By weeks 4 and 8, however, the ADSCs-SF group exhibited a remarkable and highly significant improvement in muscle mass relative to the SF group. This improvement in muscle regeneration was evident through increased muscle tissue volume and more robust cell infiltration, and the difference between the two scaffold groups persisted consistently throughout this study.

By 12 weeks, the ADSCs-SF group demonstrated the most advanced tissue regeneration, with a well-organized muscular layer that closely resembled the structure of native bladder tissue. Histological analysis (Figure 4B) revealed a highly structured muscle layer with distinct muscle fibers and cellular alignment, similar to what is seen in healthy, uninjured bladder tissue. This suggested that the incorporation of ADSCs into the scaffold significantly enhanced muscle regeneration, contributing to more effective tissue repair and integration.

In contrast, the SF group exhibited a smoother muscle layer that although present, remained less structured and histologically distinct from normal bladder muscle tissue (Figure 4A). While muscle cells were present within the SF scaffold, the tissue lacked the organization and fiber alignment characteristics of the native bladder muscle. This less mature muscle layer indicated that the SF scaffold alone, without the presence of ADSCs, was less effective in promoting full muscle regeneration and organization. Overall, the results highlighted the superior regenerative potential of ADSCs-SF scaffolds, which not only improved muscle mass but also supported the development of a more functional and anatomically similar muscle structure.

#### 3.3.3. Vessel Density

At two weeks post-implantation, both scaffold groups exhibited a significant reduction in vessel density compared to the control group (Figure 5C), indicating a delay in vascularization during the early stages of tissue integration. This early decrease in vessel density suggests that both scaffold types initially struggled to support sufficient blood vessel formation, a crucial aspect for tissue survival and regeneration.

Between weeks 4 and 8, the ADSCs-SF group demonstrated a significantly higher number of regenerated blood vessels relative to the SF group (Figure 5C). This improvement in vascularization was particularly notable as the ADSCs-SF scaffolds began to show more extensive network formation, which is essential for delivering nutrients and oxygen to the regenerating tissue. The presence of ADSCs appeared to enhance angiogenesis, promoting the growth of new blood vessels that better supported the developing tissue structure.

By 12 weeks post-implantation, the ADSCs-SF group displayed prominent, well-formed large blood vessels along the serosal surface of the scaffold (Figure 4B). These large vessels were distinct and more mature, indicating that the ADSCs-SF scaffold had effectively facilitated the establishment of a more developed vascular network. In contrast, the SF group exhibited fewer and less mature blood vessels at this stage, with less vascularization overall. Additionally, capillaries were clearly observed within the central region of the ADSCs-SF scaffolds (Figure 4B), further underscoring the superior vascularization and overall tissue integration promoted by the ADSC-based scaffold. This enhanced vessel density in the ADSCs-SF group was crucial for the overall success of the tissue regeneration process, as it likely contributed to better tissue survival, nutrient exchange, and integration with the surrounding host tissue.

#### 3.3.4. Inflammatory Cells

At 2 weeks post-implantation, both scaffold groups exhibited a significant degree of inflammatory cell infiltration beneath the urothelium (Figure 4A,B and Figure 5D), reflecting an early immune response to the implanted scaffolds. This initial inflammation is typical of tissue repair and healing processes, where immune cells are recruited to the site to clear debris and support tissue regeneration. However, the extent of this infiltration was notable in both groups, indicating that the foreign materials within the scaffolds triggered an immune reaction, despite the potential for tissue integration.

Between 4 and 8 weeks, the SF group exhibited significantly higher levels of inflammatory cell infiltration compared to the ADSCs-SF group. This indicates that the SF scaffold alone may have triggered a more intense and prolonged inflammatory response, potentially due to its material properties or its limited ability to fully integrate with the host tissue. In contrast, the ADSCs-SF group showed a notable reduction in inflammatory cell infiltration during the same period. The inclusion of adipose-derived stem cells (ADSCs) likely played a crucial role in modulating the immune response, enhancing tissue healing, and reducing inflammation, which may have contributed to improved tissue regeneration and integration.

By 12 weeks post-implantation, both scaffold groups exhibited a marked decrease in inflammatory cell infiltration, suggesting that the tissue had entered a more stable phase of healing. However, there was a significant difference between the two scaffold groups, with the ADSCs-SF group showing a much lower level of persistent inflammation. This reduction in inflammation in the ADSCs-SF group likely contributed to the more favorable tissue regeneration observed, as chronic inflammation can hinder tissue healing and regeneration by promoting fibrosis and inhibiting cellular differentiation.

In conclusion, the ADSCs-SF scaffolds were more effective at reducing inflammatory responses over time, which may have helped create a more conducive environment for tissue regeneration and integration. The SF group, while still showing signs of inflammation, did not achieve the same degree of immune modulation, highlighting the potential benefits of incorporating stem cells to improve tissue healing outcomes.

### 3.4. Urothelial Regeneration and Uroplakin III Expression in ADSCs-SF and SF Scaffolds

The regenerative capacity of the grafts in both experimental groups was assessed by staining the bladder urothelium with uroplakin III and smooth muscle cells (SMCs) with α-SMA at several key time points following implantation. Throughout this study, a noticeable difference in the expression and intensity of both α-SMA and uroplakin III was observed between the two scaffold types. In particular, the ADSCs-SF scaffold consistently showed significantly higher levels of these markers compared to the SF scaffold alone.

**Figure 6:**
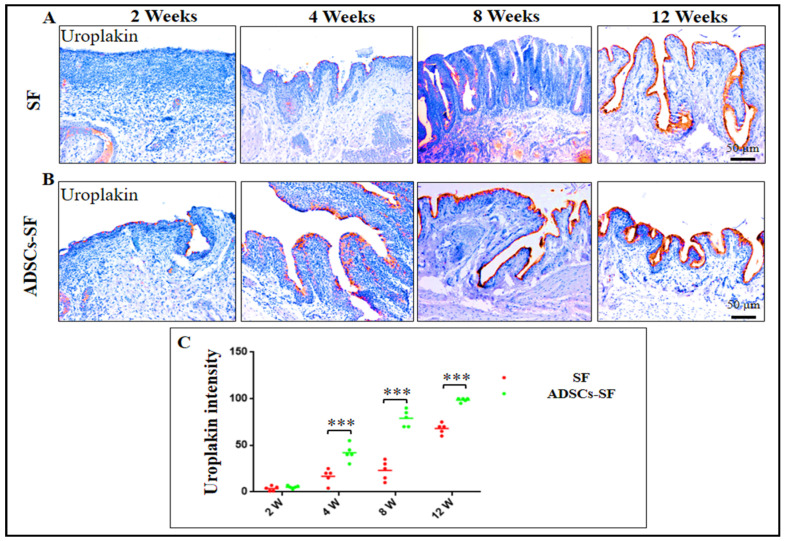
Immunohistochemical analysis of uroplakin III expression, a urothelial-specific marker, in the regenerated bladder wall at 2, 4, 8, and 12 weeks following implantation of SF and ADSCs-SF scaffolds. Panels (**A**,**B**) show the expression patterns of uroplakin III at different time points for both scaffold groups, while panel (**C**) presents the relative intensity of uroplakin III expression across these time points. Data are expressed as the mean ± SD. Statistical significance was determined using one-way analysis of variance (ANOVA) followed by Tukey’s post hoc test. Asterisks indicate statistical significance: *** *p* < 0.001.

At the two-week post-implantation mark, neither group exhibited detectable uroplakin III expression, indicating an early stage of regeneration where urothelial cells had not yet fully integrated or matured. However, starting in the fourth week and continuing through the twelfth week, a remarkable difference emerged. The ADSCs-SF group demonstrated a dramatic and sustained increase in uroplakin III intensity, significantly outperforming the SF scaffold group. This progression was visually supported by detailed imaging, as shown in Figure 6A–C, where the ADSCs-SF group exhibited a clearer, more pronounced expression of uroplakin III at each time point post-implantation.

The ADSCs-SF scaffold proved to be far superior in promoting urothelial regeneration, as evidenced by the earlier and more intense expression of uroplakin III from the fourth week onwards. This suggests that the inclusion of ADSCs within the SF scaffold significantly enhanced the regenerative capacity of the graft, facilitating more efficient and robust tissue repair compared to the SF scaffold alone.

### 3.5. Smooth Muscle Cell Regeneration in ADSCs-SF and SF Scaffolds

**Figure 7:**
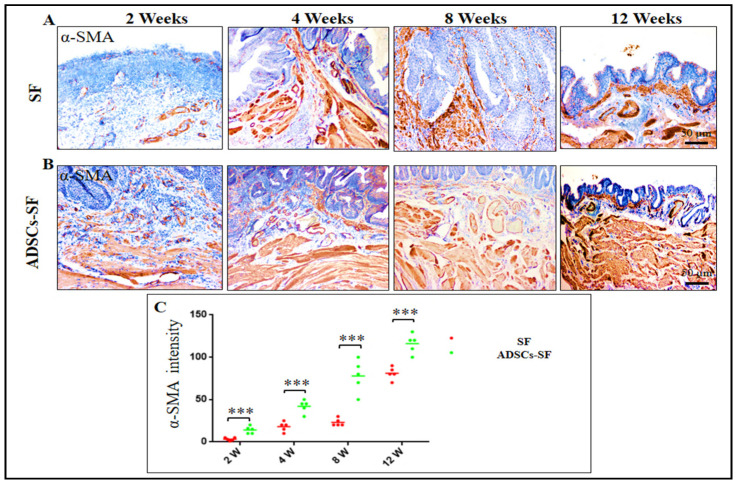
Immunohistochemical analysis of α-SMA expression, a smooth muscle cell (SMC)-specific marker, in the regenerated bladder wall at 2, 4, 8, and 12 weeks following implantation of SF and ADSCs-SF scaffolds. Panels (**A**,**B**) display the expression of α-SMA at each time point for both scaffold groups, while panel (**C**) presents the relative intensity of α-SMA expression. Data are shown as the mean ± SD, with statistical significance determined by one-way analysis of variance (ANOVA) followed by Tukey’s post hoc test. Asterisks denote significant differences: *** *p* < 0.001.

At the 2-week time point, both groups showed the lowest relative intensity of α-SMA, indicating the early stages of smooth muscle regeneration. However, a clear distinction between the groups became evident as this study advanced. The ADSCs-SF group showed a significantly higher increase in α-SMA intensity compared to the SF group. Between the 4th and 12th weeks, muscle bundles started to form gradually in both groups, as depicted in Figure 7A,B.

By the 12-week mark, the ADSCs-SF group showed not only significantly higher α-SMA expression but also a more advanced and organized muscle structure. The muscle bundles in the ADSCs-SF group were notably more structured, with well-defined transverse and longitudinal orientations, indicative of more mature smooth muscle tissue. In contrast, the SF group exhibited less well-organized muscle fibers, with lower α-SMA intensity and less defined bundle formation (Figure 7A,C). These findings underscore the enhanced regenerative potential of the ADSCs-SF scaffold in promoting smooth muscle tissue development and organization. The ADSCs-SF scaffold significantly enhances SMC regeneration, as evidenced by higher α-SMA expression and better muscle bundle organization at 12 weeks compared to the SF scaffold. This indicates superior smooth muscle development and alignment with the ADSCs-SF scaffold.

## 4. Discussion

A significant challenge in urology is the partial or complete replacement of the bladder, a procedure often necessary for treating a range of conditions, including neurogenic bladder, bladder cancer, bladder obstruction, and bladder fibrosis. These conditions can severely compromise bladder function, leading to incontinence, impaired urinary storage, and other debilitating symptoms. While traditional treatments such as urinary diversion or cystectomy are common, they do not fully restore normal bladder function or quality of life. Consequently, there is an ongoing need for innovative approaches to bladder reconstruction that can regenerate bladder tissue and restore its complex structure and function [28,29]. These challenges highlight the need for more effective strategies in bladder tissue engineering, such as the use of stem cells and bioengineered scaffolds, to promote functional regeneration and improve patient outcomes [30].

The unique combination of elasticity, strength, and biodegradability, along with its biocompatibility and minimal immunotoxicity, makes SF an attractive option for bladder tissue engineering [30,31,32]. This regenerative capability makes silk-based scaffolds a promising candidate for bladder tissue engineering and a potential solution to the limitations of traditional reconstruction methods [33]. Previous studies have shown that the use of silk sutures in bladder reconstruction has been associated with deleterious consequences including inflammation and urinary calculi [12,34]. One approach to improve the graft’s biocompatibility and reduce complications might involve seeding the implant with cells. In this study, SF and ADSCs-SF scaffolds were compared for bladder reconstruction in rats. Both scaffolds were well-tolerated, but the SF group showed graft shrinkage and incidence of bladder calculus. In contrast, the ADSCs-SF group demonstrated superior bladder morphology and no calculus formation. Histological analysis indicated that ADSCs-SF scaffolds supported better urothelial and smooth muscle regeneration, with enhanced vessel formation and reduced inflammation compared to SF scaffolds [35].

The bladder urothelium plays a critical role in maintaining the organ’s integrity by forming a specialized, impermeable barrier that prevents the passage of water, solutes, and harmful substances found in urine. This barrier is essential for protecting the underlying structures, including smooth muscle, blood vessels, and nerves, from the toxic effects of urine and other potentially damaging compounds [36].

As previously reported, when urothelial recovery is hindered in augmented bladders, it can significantly slow down or even prevent the subsequent regeneration of these underlying tissues, making effective bladder augmentation more challenging and less predictable [37].

The SF group exhibited noticeable urothelial hyperplasia between the 4th and 8th weeks of this study. This hyperplasia likely resulted from a sequence of chronic inflammatory responses, which were driven by the degradation of SF fragments over time. Chronic inflammation is known to trigger cellular changes that promote tissue growth, including the proliferation of the urothelial layer. The accelerated breakdown of SF fragments may have contributed to this heightened inflammatory environment, leading to an abnormal increase in urothelial cells. Furthermore, this hyperplastic response may reflect incomplete or abnormal maturation of the urothelium, which could be due to an imbalance in the healing process or a failure of proper tissue differentiation during regeneration. Previous studies have highlighted the relationship between inflammation and urothelial hyperplasia, suggesting that the persistent inflammatory state could hinder normal tissue development [26,27].

On the other hand, the ADSCs-SF group did not demonstrate urothelial hyperplasia. This absence of hyperplasia can likely be attributed to the anti-inflammatory properties of ADSCs. ADSCs are known for their ability to modulate the immune response, reduce inflammation, and promote tissue repair without triggering excessive cell proliferation [20]. This ability to temper the inflammatory response likely facilitated more controlled urothelial regeneration, allowing the tissue to mature more effectively without undergoing hyperplasia.

SF scaffolds have been shown to provide a suitable environment for urothelial cells to grow and repopulate the injured area. Therefore, while the ADSCs-SF group may have avoided the excessive hyperplasia seen in the SF group, both scaffolds provided a conducive environment for urothelial regeneration, albeit through different mechanisms. The comparable urothelial ingrowth in both groups could be attributed to the capacity of native urothelial cells to naturally migrate and integrate into the SF scaffold, as supported by prior research [38].

The well-formed muscle bundles observed in the ADSCs-SF group can be attributed to ADSCs and are known to have a dual role in tissue regeneration, where they not only integrate into the regenerating tissue but also release bioactive molecules that stimulate nearby cells and enhance the healing process [38]. This trophic support is crucial for stimulating cellular repair and promoting the regeneration of the smooth muscle layer in the bladder. Additionally, myogenic regeneration—where mesenchymal stem cells (MSCs) differentiate into smooth muscle cells—has been well-documented as one of the key therapeutic mechanisms of MSCs, including ADSCs.

In this study, significant neovascularization was observed in the ADSCs-SF scaffold by 12 weeks, in contrast to the SF-only scaffold. This result highlights the enhanced ability of ADSCs to stimulate angiogenesis, as supported by previous research [38,39,40]. The observed increase in neovascularization within the ADSCs-SF group underscores the synergistic effect of combining ADSCs with SF scaffolds, which enhances both cell survival and tissue regeneration. The vascularization observed in this group is essential for the long-term success of bladder reconstruction, as it ensures that the newly formed tissue receives the necessary support for sustained function and healing. Inflammation plays a complex role in bladder repair using scaffolds, with both positive and negative effects.

On one hand, inflammation is essential for initiating chemotaxis and facilitating scaffold breakdown, which is necessary for tissue remodeling. However, chronic inflammation can also hinder the regeneration process by leading to fibrosis, scarring, and even rejection of the implanted material [41]. The host immune system, particularly macrophages, plays a key role in the degradation of SF porous scaffolds, which can influence the overall success of bladder regeneration [42]. In the ADSCs-SF group, a significant reduction in inflammatory cell infiltration was observed, which suggests that the ADSCs helped mitigate the inflammatory response. This outcome can likely be attributed to the potent anti-inflammatory effects of ADSCs, which are known to modulate the immune response and reduce tissue inflammation, as discussed in earlier studies [20,43].

Uroplakin staining was used to assess the urothelial clusters, revealing that the cells exhibited characteristics consistent with a functional urothelium [44]. The presence of uroplakin-positive staining indicates the presence of mature urothelial cells, which are essential for preserving the impermeable barrier of the urothelium [44]. Therefore, the positive uroplakin staining not only confirms urothelial differentiation but also suggests that the newly formed urothelium is capable of performing its protective role effectively.

Forty-one percent of the animals in the SF group had bladder calculi. This result can be explained by the presence of foreign objects in the bladder that serve as a nidus, for the precipitation of minerals from the urine, and they are usually the cause of the development of urinary calculi. Additionally, urinary stasis and post-operative infections are also major factors in the crystallization process and the formation of stones [45]. In contrast, no bladder calculi were observed in the ADSCs-SF group, indicating a notable difference between the two groups. This absence of stone formation could be attributed to the potent anti-inflammatory properties of ADSCs. The immunomodulatory effects of ADSCs may have played a key role in reducing inflammation and preventing the development of conditions that typically promote stone formation. By modulating the inflammatory response, ADSCs may have created a more favorable environment within the urinary tract, thereby minimizing the risk of crystallization and stone formation, as evidenced by the lack of calculi in this group. These findings underscore the potential of ADSCs to mitigate adverse conditions that contribute to the formation of urinary stones.

## 5. Conclusions

One of the key findings highlighted in this study is that ADSCs-SF scaffolds not only enhance tissue regeneration but also minimize the complications associated with traditional SF scaffolds. The improved urothelial and smooth muscle regeneration, along with reduced inflammation and vascularization, highlight the potential of ADSCs-SF scaffolds for advancing bladder reconstruction techniques. Future research should continue to explore and refine the degradability of SF scaffolds, focusing on optimizing their application and addressing remaining challenges in bladder tissue engineering. This study was conducted on female rats only so future studies are also needed to evaluate if there are differences in bladder regeneration in both sexes using SF scaffolds.

## Data Availability

Data are contained within the article.
